# Coagulation Dysfunction in Patients with Liver Cirrhosis and Splenomegaly and Its Countermeasures: A Retrospective Study of 1522 Patients

**DOI:** 10.1155/2023/5560560

**Published:** 2023-06-07

**Authors:** Yunfu Lv, Ning Liu, Yejuan Li, Jincai Wu, Jinfang Zheng, Xinqiu Li, Min Zeng

**Affiliations:** ^1^Department of General Surgery, Hainan General Hospital (Hainan Medical College Affiliated People's Hospital), Haikou, 570311 Hainan Province, China; ^2^Reproductive Medicine Center of Hainan Women and Children's Medical Center, Haikou, 570206 Hainan Province, China; ^3^Department of Surgery, Renhuai People's Hospital, Zunyi, 564500 Guizhou Province, China

## Abstract

**Objective:**

Patients with cirrhosis and splenomegaly often have coagulation dysfunction which affects treatment and prognosis. This study explores the status, grading, and treatment strategies of coagulation dysfunction in patients with liver cirrhosis and splenomegaly.

**Methods:**

A retrospective cohort study was conducted on the clinical data on consecutive patients with cirrhosis and splenomegaly treated at Hainan General Hospital, China, from January 2000 to December 2020. Starting research in January 2022.

**Results:**

Among 1522 patients included into this study, 297 (19.5%) patients had normal results in all five coagulation tests (prothrombin time, prothrombin activity, activated partial thromboplastin time, thrombin time, and fibrinogen), and 1225 (80.5%) had coagulation dysfunction in at least one of these tests. There were significant differences (*P* < 0.05) in treatment efficacy on these patients for three of these five coagulation tests, with the exception of prothrombin activity and thrombin time. When coagulation dysfunction was classified into grades I, II, and III based on scores from the three significant coagulation tests, prothrombin time, activated partial thromboplastin time, and fibrinogen, significant differences in surgical outcomes were found among the three grades of coagulation dysfunction and between grades I and III (*P* < 0.05). The operative mortality rate in patients with grade III in treating liver cancer, portal hypersplenism, and/or splenomegaly was 6.5%. There was no significant difference between patients with grades I and II (*P* > 0.05).

**Conclusions:**

Approximately, 80% of patients with liver cirrhosis and splenomegaly had coagulation dysfunction. Surgery is feasible for grade I and II patients. For grade III patients, nonsurgical treatment should be given first, and surgery should only be considered when the coagulation function returns to normal or near-normal levels after treatment. This trial is registered with MR-46-22-009299.

## 1. Introduction

Patients with liver cirrhosis and splenomegaly who are assessed for surgical treatment should routinely be tested for prothrombin time (PT), prothrombin activity (PTA), activated partial thromboplastin time (APTT), thrombin time (TT), and fibrinogen (Fib) upon hospital admission [1–3] to assess disease progression and guide treatment decisions [4, 5]. If the values for these factors are abnormal, surgery is generally considered as not indicated, and these patients should be treated nonsurgically.

A normal coagulation function is important for surgical patients. Blood coagulation is triggered by two pathways: intrinsic, which includes the blood-derived coagulation factors I-XIII or FI-FXIII, and extrinsic, which includes the tissue-derived tissue factors (TFs) that initiate coagulation [6, 7]. Although the initiation of coagulation factors involved in these two pathways are not identical [8], they are not completely independent of each other. Both pathways activate factor X (FX), thereby generating thrombin and fibrin clots [9]. Moreover, some coagulation factors involved in the two pathways activate and enhance each other, thus achieving coagulation cooperatively.

PT is the time required for clotting to occur in platelet-poor plasma after the addition of excess TF, which triggers the conversion of prothrombin to thrombin. It primarily reflects the functional status of the extrinsic coagulation system, and it serves as an important indicator for monitoring oral anticoagulation therapy [10]. Prolonged PT is observed in patients with congenital coagulation factor I, II, V, VII, and X deficiency and in hypofibrinogenemia. Acquired coagulation factor deficiency is seen in conditions such as vitamin K deficiency [11, 12], severe liver diseases, hyperfibrinolysis, disseminated intravascular coagulation (DIC), oral anticoagulation [13, 14], potent antibiotic use [15, 16], and snakebite [17]. Shortened PT is seen in patients with conditions such as hypercoagulable states, thrombotic diseases, and oral contraception use. PTA has the same clinical significance as PT, but it more accurately reflects the activity of coagulation factors [18]. It is calculated using the formula PTA (%) = (normal PT − 8.7)/(patient′s PT − 8.7) × 100. Severely impaired liver function, liver failure, or chronic cholestasis can lead to decreased liver production of coagulation factors, thus resulting in prolonged PT and decreased PTA. PTA < 40% indicates early liver failure and poor prognosis [19, 20]. APTT is a common coagulation test that primarily reflects the functional status of the intrinsic coagulation system [21]. It tests for all plasma coagulation factors except factor VII. It is also an important indicator for monitoring heparin therapy. Prolonged APTT is seen in patients with conditions that cause decreased plasma factor VIII, IX, XI, and XII levels such as hemophilia A, hemophilia B, and factor XI deficiency; severe prothrombin deficiency such as liver diseases, obstructive jaundice, and hemolytic disease of the newborn; severe fibrinogen deficiency such as afibrinogenemia/hypofibrinogenemia, heparin use, and severe liver diseases; and hyperfibrinolysis such as late DIC and primary fibrinolysis. Shortened APTT is seen in hypercoagulable states due to events such as release of procoagulant substances into blood and increased activity of coagulation factors. Both prolonged PT and APTT can also be due to an inhibitor or coagulation factor deficiency [22]. For example, hereditary factor V deficiency has been shown to prolong PT and APTT to 20.3 s and 59.2 s, respectively [23], and to reduce coagulation factor V activity to 3% [24]. Also, congenital FX deficiency has been shown to prolong PT to >40 s and APTT to 65.0 s [25]. TT is the time taken for the plasma to clot after addition of standardized thrombin and primarily reflects the time needed for conversion of fibrinogen to fibrin. Prolonged TT is seen in the hyperfibrinolysis stage of DIC, afibrinogenemia/hypofibrinogenemia, abnormal hemoglobinemia, increased blood fibrin(ogen) degradation products, and increased heparin or presence of heparinoids as seen in patients with conditions such as systemic lupus erythematosus, liver diseases, and kidney diseases. Shortened TT has no clinical significance. Fib is a protein present in plasma that primarily reflects the levels of fibrinogen and is called factor I. Increased Fib is seen in patients with acute myocardial infarction, infection, malignant tumor, and cerebral thrombosis. Decreased Fib is seen in liver necrosis and cirrhosis caused by various reasons such as primary fibrinogen deficiency, consumptive hypocoagulable stage of DIC, and primary fibrinolysis.

The liver is the site where many coagulation factors are produced. After acute liver injury and with chronic liver diseases, the ability of the liver to synthesize coagulation factors can be reduced, resulting in a substantial reduction of many circulating coagulation factors [26]. Prolonged PT, APTT, and TT and decreased PTA and Fib indicate coagulation dysfunction due to impaired liver function [13].

Clinical evidence is currently lacking regarding the coagulation function status in patients with cirrhosis and splenomegaly. Furthermore, it is necessary to determine if the coagulation dysfunction status in these patients can be graded, and how the grading can be used to guide the choice of treatment and assess prognosis. This clinical study on 1522 patients was conducted to provide answers to the abovementioned clinical questions.

### 1.1. Conditions for Inclusion

1. Cases of hepatocirrhosis and splenomegaly caused by various reasons
2. The clinical data of the study were approved by the patients or their families
3. Complete collection of clinical data

### 1.2. Conditions for Exclusion

1. Nonliver cirrhosis patients with splenomegaly
2. The clinical data of the study were not recognized by the patients or their families
3. Incomplete collection of clinical data

## 2. Patients and Methods

### 2.1. Ethics Statement

All data were obtained from the database for patients treated at Hainan General Hospital, China. This study was approved by the Hospital Ethics Committee. Informed consent was obtained from all patients for all treatment procedures (please refer to the supplementary information). This study was conducted in accordance with the relevant guidelines and regulations of STROBE.

### 2.2. Patients

Consecutive patients with liver cirrhosis and splenomegaly admitted to Hainan General Hospital, China, from January 2000 to December 2020 entered into this study. Of 1522 patients, there were 1035 males (68.0%) and 487 females (32.0%), with a male-to-female ratio of 2.1 : 1. The patients' ages ranged from 14 to 80 years, with a median of 48 years. Research started in January 2022. Among them, 1233 patients (81.0%) had hepatitis B cirrhosis, 168 (11.0%) hepatitis C cirrhosis, 38 (2.5%) biliary cirrhosis, 30 (2.0%) autoimmune hepatitis cirrhosis, 30 (2.0%) alcoholic cirrhosis, and 23 (1.5%) other types of cirrhosis. All patients had nodular liver atrophy and splenomegaly based on ultrasound and CT and monolineage or multilineage peripheral cytopenias as determined by venous blood tests. All patients underwent surgery. Specifically, 156 patients (10.2%) underwent liver resection for liver cancer; 498 (32.7%) underwent devascularization of the lower esophagus and gastric fundus+splenectomy for massive gastrointestinal bleeding (≥1000 mL); 461 (30.3%) underwent splenectomy+portal-azygos disconnection for moderate or severe hypersplenism; 181 (11.9%) underwent splenectomy for hypersplenism; 10 (0.7%) underwent portacaval shunt; 6 (0.4%) underwent liver transplantation; and 210 (13.8%) underwent splenectomy for splenomegaly (defined as a spleen that extended beyond the midline of the abdomen or below the line joining the two anterior superior iliac spines) and with poor quality of life.

### 2.3. Coagulation Tests

For each of the coagulation tests, 1.8 mL of peripheral venous blood from a patient was collected into a tube containing 0.2 mL of 109 mmol/L sodium citrate. The collected blood was then sent to the hospital laboratory for testing within 1 hr using the tube test method after shaking.

### 2.4. Retrospective Cohort Study

The study was conducted first by designing a study form to collect data from patients. Patients were excluded from this study if they had no cirrhosis and splenomegaly, did not undergo surgery to treat these conditions or their complications, or had incomplete data. The proportions of patients with cirrhosis and splenomegaly with normal and abnormal coagulation tests were then determined. The relationship between the different levels of the five coagulation parameters—PT, PTA, APTT, TT, and Fib—with surgical outcomes and the impact of the Child-Pugh classification on coagulation factors were analyzed. Finally, the coagulation dysfunction was scored and graded, and the relationship between the grading with surgical outcomes was analyzed with the aim of using the different grading to guide treatment options to improve perioperative management.

### 2.5. Statistical Analysis

All data were analyzed using SPSS 25.0. Measurement data were expressed as median (P25, P75) and compared using the rank sum test or chi-square test. Count data were expressed as percentages. The relationship between the Child-Pugh classification and the gradings for different coagulation tests was analyzed using the Student's *t*-test. The *F*-test was used for the analysis of variance. The relationship between gradings of coagulation dysfunction and surgical outcomes was analyzed using the chi-square test. A *P* < 0.05 was considered statistically significant.

## 3. Results

### 3.1. Proportions of Patients with Cirrhosis and Splenomegaly with Normal and Abnormal Coagulation Tests

The determination of normal and abnormal results for each of the five coagulation tests used in this study is shown in Table 1.

**Figure 1:**
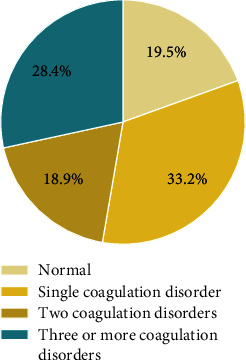
Proportions of patients with normal and abnormal coagulation tests. It includes the proportion of patients with normal coagulation function, the proportion of patients with only one kind of coagulation abnormality, the proportion of patients with two kinds of coagulation abnormalities, and the proportion of patients with three or more kinds of coagulation abnormalities.

Eighty percent of the 1522 patients had at least one abnormal coagulation test. The proportions of patients with normal and abnormal coagulation tests are shown in Figure 1.

### 3.2. Types of Coagulation Disorders and Surgical Outcomes

The types of coagulation disorders in 1225 patients (80.5% of all patients) and their surgical outcomes are shown in Table 2. There was no significant difference in therapeutic efficacy between patients with one, two, and three or more coagulation disorders (*χ*^2^ = 0.267, *P* > 0.05).

### 3.3. Comparison of Postoperative Efficacy with the Five Coagulation Parameters

Effective refers to the disappearance of clinical symptoms and signs. No change indicates no improvement after treatment relative to before treatment. Death refers to death during hospitalization or within two weeks after the patient requested discharge. Comparison of postoperative efficacy with the five coagulation parameters tested revealed significant differences for PT, APTT, and Fib (*P* < 0.05), but not for PTA and TT (*P* > 0.05) (Table 3).

### 3.4. Influence of the Child-Pugh Classification on Coagulation Tests

**Figure 2:**
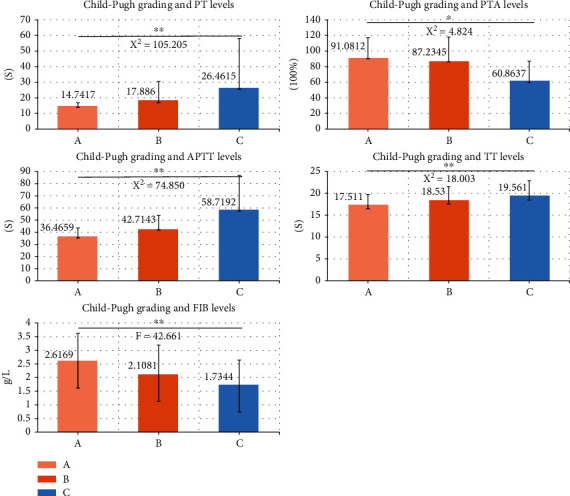
The Child-Pugh grading and five coagulation functions. The Child-Pugh classification of liver function (A, B, C) was significantly correlated with coagulation function items PT, PTA, APTT, TT, and FIB (*P* < 0.05). The worse the liver functions, the more abnormal these coagulation items were present (note: ^∗^*P* < 0.05; ^∗∗^*P* < 0.001).

The relationship between the Child-Pugh classification and coagulation test is shown in Figure 2. Significant differences were found for all the coagulation tests with the Child-Pugh classification (*P* < 0.05 or 0.001).

### 3.5. Coagulation Dysfunction Grading

Among patients with coagulation dysfunction, only 41.3% had one coagulation disorder, and 58.7% had more than one coagulation disorder. Given the significant differences in efficacy with the factors PT, APTT, and Fib (Table 2), these three parameters were used to score and grade coagulation dysfunction to distinguish the severity levels of coagulation dysfunction and to investigate the influence of coagulation dysfunction grading on the choice of treatment (Table 4).

### 3.6. Coagulation Dysfunction Grading and Surgical Outcomes

**Figure 3:**
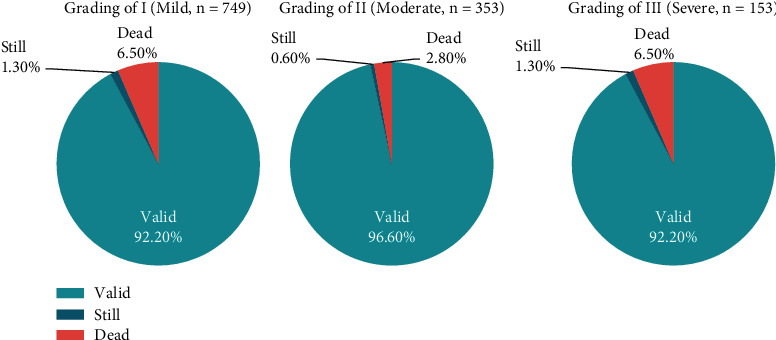
Coagulation dysfunction grading and surgical outcome. There were significant differences (*P* < 0.05) in surgical treatment outcomes (effective, ineffective, and dead) for grades I, II, and III coagulation disorders. The worse the grade (more), the higher the mortality rate, of which the mortality rate of grade III is 6.5%, and special attention should be paid.

Using the grading criteria of coagulation disorders in Table 4, of the 1255 patients with coagulation disorders, 749 patients (59.7%) were in grade I (mild), 353 patients (28.1%) in grade II (moderate), and 153 patients (12.2%) in grade III (severe). Their association with surgical treatment outcomes (valid, still, and dead) is shown in Figure 3.

## 4. Discussion

Although there are many factors that affect surgical outcomes of patients with cirrhosis and splenomegaly [27], coagulation function is a factor that cannot be ignored [28]. This study found that approximately 20% of patients with liver cirrhosis and splenomegaly had normal coagulation function, whereas 80% had coagulation dysfunction. The liver plays a central role in coagulation [29] as it is the main source of synthesizing many proteins that are crucial for the normal function of the coagulation cascade. Therefore, liver diseases, especially cirrhosis, can lead to abnormal coagulation [26, 30]. The Child-Pugh score is the gold standard for assessing liver function [31]. The levels of PT, APTT, and TT increased significantly from Child-Pugh's level B, while PTA and FIB decreased significantly from level B. The lower the grading of the Child-Pugh, the more severe the coagulation dysfunction becomes, which is similar to the results of Caterina et al. [32] Only cirrhotic patients with good liver function can safely undergo surgery; conversely, surgery can lead to a high mortality in patients with advanced cirrhosis [33]. As a consequence, in addition to the rational selection of treatment, effective medications to improve coagulation dysfunction should also be given to these patients in perioperative management.

In addition, the effect of blood ammonia cannot be ignored. The increase in blood ammonia in patients with liver cirrhosis is due to impaired liver function during cirrhosis. Therefore, blood ammonia cannot be synthesized into urea and excreted through the kidneys, resulting in blood ammonia accumulation. Tarantino et al. [34] studied 153 patients with liver cirrhosis of different causes and found that the lower the Child-Pugh grading is the higher the blood ammonia content increases. Elevated blood ammonia levels can easily lead to hepatic encephalopathy [35, 36], which indirectly reflects a decrease in liver function. The numerous clotting products synthesized in the liver can become severely impaired, leading to bleeding. If unexplained upper gastrointestinal bleeding is accompanied by elevated blood ammonia, more than 90% of patients may be caused by liver cirrhosis and decompensation of liver function. Elevated blood ammonia is an independent predictor of liver disease-related complications and mortality [37]. For such patients, routine blood ammonia testing should be performed, and any abnormalities should be promptly addressed.

Nakai et al. [11] reported that a 73-year-old patient with iron deficiency anemia and prolonged prothrombin time was confirmed by laboratory examination. After vitamin K supplementation, his anemia, nutritional status, serum vitamin K level, and prothrombin time were continuously corrected. When Owen and Bowie [12] administered single or multiple doses of warfarin to rats, the levels of prothrombin and factors VII, IX, and X decreased, while factors V, VIII, XI, and XII also decreased moderately. After taking vitamin K1, the levels of all eight factors quickly returned to normal.

Among patients with coagulation dysfunction, 42% had one coagulation disorder, and approximately 58% had more than one coagulation disorder, including 23% with two coagulation disorders and 35% with three or more coagulation disorders. Although there was no significant difference in the overall efficacy among the three groups, significant differences in efficacy were found between the different levels for three of the five coagulation parameters, namely, PT, APTT, and Fib (*P* < 0.05). These findings demonstrated the significant impact of these three parameters on surgical outcomes. To further look into the impact of the various coagulation parameters on the choice of treatments and surgical outcomes, coagulation dysfunction was classified into three grades based on a scoring system developed in this study.

The scoring and grading were based on statistical differences between the groups as shown in Table 2 by taking into account the coagulation pathways that each of these factors represents. PT represents the extrinsic coagulation system, and APTT represents the intrinsic coagulation system. Fib can be converted into fibrin, which is a protein that spontaneously polymerizes to form a hemostatic clot with a fibrin network that reinforces coagulation, much like the steel bars in concrete. However, Fib in this study was not assigned a score of 5 because surgical mortality in patients with Fib < 1 g/L was low (Table 3). It is worth noting that the scoring and grading according to the three coagulation parameters used in this study can comprehensively and accurately reflect the actual coagulation dysfunction. Moreover, it is simple and easy to implement in clinical practice. There was no statistical difference between different levels of PTA and TT, indicating that they had no significant effect on surgical outcomes. Moreover, changes in PTA levels are primarily related to PT, and changes in TT levels are primarily related to Fib. As PT and Fib are included in the scoring and grading in this study and they indirectly represent PTA and TT, it is reasonable not to include PTA and TT in the scoring and grading analysis.

There were significant differences in the surgical outcomes among the three grades of coagulation dysfunction (*P* < 0.05). In particular, efficacy was significantly lower in grade III than in grade I. Based on these results, patients with grade I coagulation dysfunction are at low surgical risks and can undergo surgery if needed. Patients with grade II coagulation dysfunction are at moderate surgical risks, indicating that nonsurgical treatment is preferred, but surgery can still be performed if necessary. Patients with grade III coagulation dysfunction are at high surgical risks, and only nonsurgical treatment options should be given. For grade III coagulation dysfunction, nonsurgical treatment should be performed. If necessary, surgery should be considered only after a blood transfusion of fresh blood or plasma, cryoprecipitation, vitamin K1, and other treatment, and blood coagulation returns to normal or close to normal level.

In 1980, the World Health Organization recommended laboratories to use an international normalized ratio (INR), which is the ISI power of the ratio of patient's measured PT to normal control PT, for oral anticoagulant monitoring to eliminate the adverse effects of oral anticoagulants; this recommendation is included in the dose plan of patients receiving warfarin [38, 39]. The normal range of INR is 0.8–1.3, which is equivalent to a PT of 11–17 s with essentially equal clinical significance. INR is primarily used to monitor oral anticoagulants, especially warfarin and antiplatelet agents. However, it is not as convenient as PT for the selection of patients and safety assessment of surgery [40] and is not as practical as our coagulation dysfunction grading.

To assess bleeding and coagulation, careful evaluation by including a history of bleeding, complete blood count, and basic coagulation tests should be performed before surgery to improve the safety of surgery and reduce the risk of intraoperative bleeding [2]. Special attention should be paid to detect coagulation dysfunction, and correction should be made when necessary. Surgical indications and contraindications should be carefully considered. Attention should also be paid to the platelet count. Safety can only be improved if these two factors—platelet count and coagulation dysfunction—are considered together [41]. The normal bleeding and coagulation mechanisms of the body are primarily achieved by the integrity of the capillary wall, the quantity and quality of platelets, and the coagulation function [2, 3, 41, 42]. Furthermore, platelets play an important role in hemostasis [3, 43]. When tissue is damaged, platelets first release vasoconstrictor substances, such as epinephrine, serotonin, and catecholamine, to promote vasoconstriction. Meanwhile, platelets adhere to and accumulate at the injury site of the blood vessel, forming a platelet clot, thereby achieving initial hemostasis [43, 44]. Platelets can also activate the coagulation system and convert plasma fibrinogen into fibrin to form a fibrin network to strengthen the clot. Additionally, platelets nourish and support capillaries and maintain the integrity of the vessel wall. Decreased levels or impaired function of platelets can cause bleeding; in fact, prolonged bleeding time is a reliable indicator of platelet function in vivo [45], and surgical outcomes can vary significantly with the degrees of platelet reduction [46]. Platelet count was not included in our coagulation dysfunction scoring and grading system because coagulation dysfunction and platelet count can lead to contradictory surgical recommendations. The purpose of coagulation dysfunction grading is to evaluate the condition and guide treatment such that surgery should not be performed when the score is high. Conversely, surgery should not be considered for patients with low platelet counts, whereas surgical treatment is recommended for patients with normal/high platelet counts. For example, a high platelet count ranging from 99 × 10^9^/L to 50 × 10^9^/L could theoretically be assigned a score of 1 based on this scoring system, indicating that surgery could be an option; however, in actuality, if the coagulation dysfunction is severe, surgery should still not be recommended, and nonsurgical treatment should be given. A low platelet count < 30 × 10^9^/L would theoretically be assigned a score of 5 based on this scoring system, indicating that surgery is contraindicated. However, the cause of the platelet reduction would need to be considered because restoration of the platelet count to normal would be possible by performing surgery to remove an enlarged spleen [47, 48]. Therefore, we did not include platelet reduction in the scoring and grading of coagulation dysfunction. We believe it is better to develop a coagulation dysfunction grading system separately and consider decreased platelet counts as another equally important element in surgical risk assessment.

There are many methods to evaluate blood coagulation function [20, 36]. This article puts forward our view from a clinical point of view. There may be some deficiencies for reference only.

## Figures and Tables

**Table 1:** Normal and abnormal values of 5 coagulation indicators.

Normal range	Abnormal
PT 9-15 s	PT > 18 s
PTA 70-100%	PTA < 69%
APTT 21-40s	APTT > 40s
TT 6-215	TT > 21 s
Fib 2-4 g/L	Fib < 2 g/L

**Table 2:** Types of coagulation disorders and treatment outcomes in patients with cirrhosis and splenomegaly.

Group	Number of coagulation disorders	Types of coagulation disorders	Number of patients, *n* (%)	Treatment results	
				Effective, *n* (%)	Dead, *n* (%)
1	One	PT (>15 s)	124	122	2 (1.6)
		APTT (>40 s)	41	41	0 (0.0)
		FIB (<2 g/L)	305	289	16 (5.2)
		TT (>21 s)	36	34	2 (5.6)
		Subtotal	506 (41.3)	486 (96.0)	20 (4.0)

2	Two	PT+APTT	93	89	4 (4.3)
		PT+PTA	68	65	3 (4.4)
		PT+FIB	91	85	6 (6.6)
		PT+TT	16	15	1 (6.3)
		FIB+APTT	8	8	0
		FIB+TT	8	8	0
		APTT+TT	3	3	0
		Subtotal	287 (23.4)	273 (95.1)	14 (4.9)

3	Three or more	PT+APTT+FIB	30	27	3 (10.0)
		PT+FIB+TT	7	7	0
		PT+APTT+TT+PTA	6	5	1 (16.6)
		FIB+APTT+TT	5	5	0
		PT+APTT+FIB+PTA	235	230	5 (2.1)
		PT+FIB+APTT+TT	9	8	1 (11.1)
		PT+FIB+APTT+TT	140	130	10 (7.1)
		Subtotal	432 (35.3)	412 (95.8)	20 (4.6)

Abbreviations: APTT: activated partial thromboplastin time; Fib: fibrinogen; PT: prothrombin time; PTA: prothrombin activity; TT: thrombin time.

**Table 3:** Comparison of postoperative treatment outcomes of each group for the five coagulation tests.

Coagulation test	Test value	Number of patients, *n* (%)	Treatment results, *n* (%)		
			Effective	Ineffective	Dead
PT (s) *n* = 1522	<30	1484 (97.4)	1385 (93.3)	47 (3.2)	52 (3.5)
	30-40	19 (1.3)	15 (78.9)	1 (5.3)	3 (15.8)
	>40	19 (1.3)	12 (63.1)	1 (5.3)	6 (31.6)
	*H* value		12.8		
	*P* value		0.005		

PTA (%) *n* = 1212	>55	1056	963 (91.2)	49 (4.6)	44 (4.2)
	55-40	78	70 (89.8)	4 (5.1)	4 (5.1)
	<40	76	66 (86.8)	5 (6.6)	5 (6.6)
	*H* value		5.634		
	*P* value		0.060		

APTT (s) *n* = 1522	<55	1347 (88.5)	1300 (96.5)	0 (0.0)	47 (3.5)
	55-65	142 (9.3)	138 (97.2)	0 (0.0)	4 (2.8)
	>65	33 (2.2)	27 (81.8)	1 (3.0)	5 (15.2)
	*H* value		16.801		
	*P* value		<0.001		

FIB (g/L) *n* = 1646	>2	578 (35.1)	552 (95.5)	2 (0.3)	24 (4.2)
	2-1	989 (60.1)	954 (965)	4 (0.4)	31 (3.1)
	<1	79 (4.8)	75 (94.9)	0 (0.0)	4 (5.1)
	*H* value		20.6		
	*P* value		0.001		

TT (s) *n* = 1605	<21	1162 (72.4)	1122 (96.6)	0 (0.0)	40 (3.4)
	21-35	430 (26.8)	410 (95.3)	0 (0.0)	20 (4.7)
	>35	13 (0.8)	11 (84.6)	0 (0.0)	2 (15.4)
	*H* value		5.386		
	*P* value		0.055		

Abbreviations: APTT: activated partial thromboplastin time; Fib: fibrinogen; PT: prothrombin time; PTA: prothrombin activity; TT: thrombin time.

**Table 4:** Coagulation dysfunction grading.

Score	1	2	3	4	5
PT (s)	19–25	26–30	31–35	36–40	>40
APTT (s)	41–50	51–55	56–60	61–65	>65
Fib (g/L)	1.9–1.5	1.4–1.0	0.9–0.5	<0.5	0

Note: a score of ≤2 is grade I (mild), 3–4 is grade II (moderate), and ≥5 is grade III (severe). Abbreviations: PT: prothrombin time; APTT: activated partial thromboplastin time; Fib: fibrinogen.

## Data Availability

All data generated or analyzed during this study are included in this published article.

## Abbreviations

AISF: Association for the study of liver diseases
APTT: Activated partial thromboplastin time
DIC: Disseminated intravascular coagulation
FIB: Fibrinogen
FX: Factor X
INR: International normalized ratio
PT: Prothrombin time
PTA: Prothrombin activity
SIMI: Society of Internal Medicine
TFs: Tissue factors
TT: Thrombin time.
